# Effect of supplementation of allicin on methanogenesis and ruminal microbial flora in Dorper crossbred ewes

**DOI:** 10.1186/s40104-015-0057-5

**Published:** 2016-01-15

**Authors:** Tao Ma, Dandan Chen, Yan Tu, Naifeng Zhang, Bingwen Si, Kaidong Deng, Qiyu Diao

**Affiliations:** Feed Research Institute, Chinese Academy of Agricultural Sciences, Key Laboratory of Feed Biotechnology of the Ministry of Agriculture, Beijing, 100081 China; College of Animal Science, Jinling Institute of Technology, Nanjing, Jiangsu 210038 China

**Keywords:** Allicin, Digestibility, Ewe, Methane, Microbial flora

## Abstract

**Background:**

Garlic extracts have been reported to be effective in reducing methanogenesis. Related mechanisms are not well illustrated, however, and most studies have been conducted *in vitro*. This study investigates the effects of supplementary allicin (AL) in sheep diet on *in vivo* digestibility, rumen fermentation, and shifts of microbial flora.

**Methods:**

Two experiments were conducted using Dorper × thin-tailed Han crossbred ewes. In experiment 1, eighteen ewes (60.0 ± 1.73 kg BW) were randomly assigned for 29 days to either of two dietary treatments: a basal diet or the basal diet supplemented with 2.0 g AL/head·day to investigate supplementary AL on nutrient digestibility and methane emissions. In experiment 2, six ewes (65.2 ± 2.0 kg BW) with ruminal canulas were assigned to the same two dietary treatments as in experiment 1 for 42 days to investigate supplementary AL on ruminal fermentation and microbial flora. The methane emissions were determined using an open-circuit respirometry system and microbial assessment was done by qPCR of 16S rRNA genes.

**Results:**

Supplementary AL increased the apparent digestibility of organic matter (*P* < 0.001), nitrogen (*P* = 0.006), neutral detergent fiber (*P* < 0.001), and acid detergent fiber (*P* = 0.002). Fecal nitrogen output was reduced (*P* = 0.001) but urinary nitrogen output was unaffected (*P* = 0.691), while nitrogen retention (*P* = 0.077) and nitrogen retention/nitrogen intake (*P* = 0.077) tended to increase. Supplementary AL decreased methane emissions scaled to metabolic bodyweight by 5.95 % (*P* = 0.007) and to digestible organic matter intake by 8.36 % (*P* = 0.009). Ruminal pH was unaffected (*P* = 0.601) while ammonia decreased (*P* = 0.024) and total volatile fatty acids increased (*P* = 0.024) in response to supplementary AL. Supplementary AL decreased the population of methanogens (*P* = 0.001) and tended to decrease that of protozoans (*P* = 0.097), but increased the populations of *F. succinogenes* (*P* < 0.001), *R. flavefaciens* (*P* = 0.001), and *B. fibrisolvens* (*P* = 0.001).

**Conclusions:**

Supplementation of AL at 2.0 g/head·day effectively enhanced OM, N, NDF, and ADF digestibility and reduced daily methane emissions (L/kg BW^0.75^) in ewes, probably by decreasing the population of ruminal protozoans and methanogens.

## Background

Methane has been proven the second-most anthropogenic greenhouse gas [1] because of its concentration in the atmosphere and its global warming potential is 21 times that of carbon dioxide [2]. Domestic ruminants have been blamed for substantially contributing to methane emissions. It would be of great value to decrease methane emissions, as methane production in ruminants represents a loss of about 2–15 % of feed energy [3]. In addition, limiting methane emissions from ruminants is not only beneficial for environmental protection, but also has potential economic benefits that could be derived from the application of carbon trading markets [4].

Numerous chemical additives to ruminant feed have been used to inhibit methane emissions. These chemicals, however, are either toxic to hosts or exhibit only transient effects on methanogenesis [5] and so-called ‘natural products’ seem to be more acceptable to consumers. Plants that contain bioactive products, such as essential oils, saponins, and tannins, can protect themselves against microbial and insect attack [6].

Allicin (AL) is one of the active components of garlic (*Allium sativum*); it has a variety of antimicrobial activities [7]. Studies of the effect of AL on methane emissions are still limited and previous studies focused mainly on the effect of other garlic components, such as garlic oil [8], garlic powder [9], and diallyl disulfide (DADS) [10], on nutrient digestibility and methane emissions by sheep and cows. Although it is generally accepted that those supplements’ activities relate to altering microbial fermentation or flora in the rumen, related mechanisms could be different. Microscopy used to be a key method in microbial quantification, and although this method allows one to determine the total number of microorganisms accurately, it has almost no capacity to distinguish among different species of bacteria [11]. Real-time quantitative PCR (q-PCR) methods can help overcome this problem and allow one to quantify specific bacteria or groups of microorganisms accurately. This study therefore investigated the effect of AL on ruminal fermentation, digestibility, and populations of protozoans, methanogens, and four cellulolytic bacteria in the rumen by using a q-PCR technique based on the 16S rRNA gene. We hypothesized that supplementary AL could reduce the population of protozoans and methanogens, but might have different effects on cellulolytic bacteria.

## Methods

This study was conducted from March 2013 to May 2013 at the Experimental Station of the Chinese Academy of Agricultural Sciences (CAAS), Beijing, China. The experimental procedures were approved by the Animal Ethics Committee of CAAS, and humane animal care and handling procedures were followed throughout the experiment.

### Animals, treatments, and experimental procedure

#### Experiment 1

Eighteen primiparous Dorper × Thin-tailed Han crossbred ewes (60.0 ± 1.73 kg BW), 12 months of age, were randomly assigned to either of two dietary treatments: a basal diet or the basal diet supplemented with allicin (AL) at 2.0 g/head·day (extracted from underground bulbs of garlic, Xi’an Feida Bio-Tech Co., Ltd., Shanxi, China). The basal diets included pelleted total mixed rations (TMR) and Chinese wild rye hay (Table 1); in the experimental diet, allicin was mixed with pelleted TMR. The ewes were fed 1500 g pelleted TMR at 0800 h and 200 g of Chinese wild rye hay at 1200 h daily. This feeding level was formulated to meet the maintenance and growth requirements of yearling ewes (60 kg BW) according to NRC (2007) [12]. All animals were housed in individual pens and had free access to fresh water over the experimental period.

**Table 1:** Ingredients and chemical compositions of experimental diets (% of DM)

Item^b^	Total mixed ration	Chinese wildrye hay
Ingredient, % of DM		
Corn	17.0	
Soybean meal	12.0	
Chinese wildrye hay	68.7	
CaHPO_4_	1.35	
Limestone	0.25	
NaCl	0.50	
Premix^a^	0.24	
Chemical composition (deteremined)		
DM (% as fed)	88.6	91.4
OM,	80.8	90.6
GE, MJ/kg of DM	17.2	17.6
CP	12.2	8.50
NDF	41.4	70.7
ADF	21.8	38.1

^a^Manufactured by Precision Animal Nutrition Research Centre, Beijing, China. The premix contained (per kg): 22.1 g Fe, 2.25 g Cu, 9.82 g Mn, 27.0 g Zn, 0.19 g Se, 0.54 g I, 0.09 g Co, 3.2 g Vitamin A, 0.8 g Vitamin D_3_, and 0.4 g Vitamin E

^b^DM dry matter, OM organic matter, GE gross energy, CP crude protein, NDF neutral detergent fiber, ADF acid detergent fiber

All ewes were moved into metabolism crates after a 14-day adaptation to diets and after another 7-day adaptation to metabolism crates; the amount of feed offered, refused, and feces were weighed daily and homogenized. A 10 % sample was collected during an 8-day collection period as described by Ma et al. [13]. Urine was collected daily in buckets containing 100 mL of 10 % (v/v) H_2_SO_4_. The volume was measured and a sample (10 mL/L of total volume) was collected and stored at −20 °C until analysis. Samples of feed, ort, feces, and urine were pooled to form a composite sample for each ewe.

Ruminal methane production was measured using an open-circuit respirometry system (Sable Systems International, Las Vegas, NV, USA) with three metabolism cages, each fitted with a polycarbonate head box. Measurements of methane production were staggered because only three measurement units were available. On days 0, 2, 4, and 6 of each 8-day collection period, the ewes were moved in sequence from their metabolism cages to metabolism cages equipped with head boxes for digestibility assays and methane output assessments. After a 24 hour adaptation period, individual methane production was measured over a 24 hour period as described by Deng et al. [14]. All ewes had been previously trained for confinement in head boxes attached to metabolism cages.

#### Experiment 2

Six ruminally cannulated Dorper × Thin-tailed Han crossbred ewes (65.2 ± 2.0 kg BW) were divided into two groups of three each according to crossover design and fed either of the following diets: basal diet or basal diet supplemented with allicin (AL, 2.0 g/head·day). Composition of the basal diets and the experimental regime were the same as in Experiment 1. The experiment lasted for 42 days, which consisted of two periods lasting 21 days, including 7 days of adaptation. On days 16 and 37, two 50mL samples of ruminal digesta were collected from rumen cannula using a syringe attached to a plastic tube (20-mm internal diameter), at 0, 1, 3, 6, and 9 h after the morning feeding for the measurements of ruminal fermentation parameters and microbial flora populations. The pH was measured immediately using a pH meter (Model PB-10, Sartorius Co., Goettingen, Germany) and all samples were frozen in liquid nitrogen within 5 min and then stored at −80 °C until needed.

### Analytical procedures

Dry matter (DM) content was measured by drying samples in an air-forced oven at 135 °C for 2 h (method 930.15; AOAC, 1990) [15]. Ash content was measured by placing samples into a muffle furnace at 550 °C for 5 h (method 938.08; AOAC, 1990) [15]. Organic matter (OM) was measured as the difference between DM and the ash content. Nitrogen (N) was measured according to the methods of Kjeldahl, using Se as a catalyst. Crude protein (CP) was calculated as 6.25 × N. Gross energy (GE) was measured using a bomb calorimeter (C200, IKA Works Inc., Staufen, Germany). Ether extracts (EE) were measured by weight loss of the DM on extraction with diethyl ether in Soxhlet extraction apparatus for 8 h (method 920.85; AOAC, 1990) [15]. Neutral-detergent fiber (NDF) and acid-detergent fiber (ADF) were measured according to Van Soest et al. [16] and Goering and Van Soest [17], respectively. NDF was measured without a heat stable amylase and expressed inclusive of residual ash. Ruminal VFA was measured according to the procedure described by Ma et al. [18] and ammonia N was assessed according to Broderick and Kang [19].

Total DNA from rumen fluid was extracted according to a bead-beating method as described by Zhang et al. [20]. The microbial cells were resuspended in a lysis buffer in tubes containing zirconium beads, which were then bead-beaten at 4600 rpm for 3 min in a mini-bead beater (MM400, Retsch, Hann, Germany) followed by phenol-chloroform extraction [21]. After centrifugation of the sample at 14,000 × g for 15 min at 4 °C, the supernatant was mixed with a glass milk kit (Gene Clean II kit, ZZBio Co., Ltd, Shanghai, China) and washed before a final elution step to release the DNA from the glass milk.

The amplifying primer of microbial flora, including total bacteria, methanogens, protozoans, *F. succinogenes*, *R. albus*, *R. flavefaciens*, and *B. fibrisolvens* are listed in Table 2 as described by Denman and McSweeney [22]. All primers were verified by sequencing and melting-curve analysis using a C1000™ thermal cycler and bundled software CFX96 Manager™ software version 2.1 (Bio-Rad laboratories, Inc., Hercules, CA, USA). The PCR products were purified by gel extraction and ligated into the pGM-T vector (Promega) and the recombinant plasmids were extracted using a plasmid minikit (Omega) according to the manufacturer’s instructions and quantified by A_260_ measurements. Standard curves for microbes were generated with 10^1^–10^7^ copies of recombinant plasmids per μL. The qPCR was performed using SsoFast EvaGreen Supermix (Bio-Rad), a C1000™ thermal cycler qPCR detection system, and genomic DNA as the template. All PCR amplifications used the following thermal cycling: 95 °C for 10 min, followed by 40 cycles of 94 °C for 20 s, 60 °C for annealing, extension, and collection of fluorescent signals. All samples were prepared from the ewes and each sample was assayed in triplicate.

**Table 2:** Primers for qPCR assay

Target species	Primer sequence (5’→3’)^a^	Amplicon
Total bacteria	F: CGGTGAATACGTTCYCGG	123
	R: GGWTACCTTGTTACGACTT	
Methanogens	F: TTCGGTGGATCDCARAGRGC	140
	R: GBARGTCGWAWCCGTAGAATCC	
Protozoans	F: GCTTTCGWTGGTAGTGTATT	223
	R: CTTGCCCTCYAATCGTWCT	
*F. succinogenes*	F: GTTCGGAATTACTGGGCGTAAA	121
	R: CGCCTGCCCCTGAACTATC	
*R. flavefaciens*	F: GATGCCGCGTGGAGGAAGAAG	286
	R: CATTTCACCGCTACACCAGGAA	
*R. albus*	F: GTTTTAGGATTGTAAACCTCTGTCTT	270
	R: CCTAATATCTACGCATTTCACCGC	
*B. fibrisolvens*	F: TAACATGAGAGTTTGATCCTGGCTC	135
	R: CGTTACTCACCCGTCCGC	

^a^Primers were designed according to Denman and McSweeney [22]

### Statistical analyses

The data on digestibility and nitrogen balance were analyzed by the independent sample *t*-test. Data referring to ruminal fermentation parameters and microbial flora measured at each sampling time were analyzed using repeated measures data of ANOVA. All statistical analyses were performed by using SPSS (SPSS Inc., Chicago, IL, USA) and significant differences were accepted if *P* < 0.05.

## Results

Supplementation of AL increased apparent digestibility of OM (*P* < 0.001), N (*P* = 0.006), NDF (*P* < 0.001), and ADF (*P* = 0.002) (Table 3). Daily fecal N output decreased from 10.7 to 9.34 g/d (*P* < 0.001) while urinary N output was unaffected (*P* = 0.691). Although no significant effect was observed, either N retention or the ratio of N retention/N intake tended to increase (*P* = 0.071) when AL was added.

**Table 3:** Effects of supplementary allicin (AL) on the apparent digestibility of nutrients and nitrogen balance in ewes

Item^a^	Treatments^b^		SEM	*P* value
	Basal diet	AL		
DM intake, g/d	1,512.4	1,512.4	0.029	0.524
Apparent digestibility, %				
OM	60.3	67.9	1.07	<0.001
N	66.6	70.9	0.86	0.001
NDF	37.9	51.8	1.90	<0.001
ADF	38.8	50.5	2.14	0.001
Fecal N, g/d	10.7	9.34	0.39	0.001
Urinary N, g/d	14.9	14.5	0.87	0.691
N retention, g/d	6.54	8.30	0.95	0.071
N retention/N intake, %	20.3	25.8	2.10	0.071

^a^DM dry matter, OM organic matter, N nitrogen, NDF neutral detergent fiber, ADF acid detergent fiber

^b^CON ewes fed basal diet, AL ewes fed basal diet supplemented with allicin

Supplementation of AL had no significant effect on daily methane output by ewes (*P* > 0.05), but decreased daily methane output from 2.85 to 2.69 L/kg BW^0.75^ (*P* = 0.007) (Table 4). In addition, daily methane output decreased from 66.1 to 61.0 l (*P* = 0.009) when scaled to DOM intake by supplementary AL. Ruminal pH was similar for both treatments (*P* = 0.601). Ammonia decreased from 10.9 to 9.37 mg/dL (*P* = 0.024) while total VFA increased from 109.4 to 125.1 mmol/L (*P* = 0.014) by supplementation of AL. The molar proportion of acetate decreased from 72.2 to 69.7 % (*P* = 0.023), while that of isobutyrate and butyrate increased from 1.32 to 1.86 % (*P* = 0.011) and from 9.43 to 11.0 % (*P* = 0.003), respectively, by supplementation of AL. No difference was observed in molar proportions of propionate (*P* = 0.155), valerate (*P* = 0.363), and the ratio of acetate to propionate (*P* = 0.455). Supplementary AL tended to increase the molar proportion of isovalerate (*P* = 0.054).

**Table 4:** Effects of supplementary allicin (AL) on daily methane production and ruminal fermentation in ewes

Item^a^	Treatments^b^		SEM	*P* value
	Basal diet	AL		
Methane production				
L	61.6	64.0	3.46	0.151
L/kg BW^0.75^	2.85	2.69	0.06	0.007
L/kg DOM intake	66.1	61.0	2.02	0.009
pH	5.98	5.96	0.04	0.601
Ammonia, mg/100 mL	10.9	9.37	0.30	0.024
Total VFA, mmol/L	109.4	125.1	4.15	0.014
Molar proportions, %				
Acetate	72.2	69.7	0.59	0.023
Propionate	14.8	14.9	0.42	0.906
Isobutyrate	1.32	1.86	0.09	0.011
Butyrate	9.43	11.0	0.24	0.003
Isovalerate	1.37	1.68	0.08	0.054
Valerate	0.89	0.71	0.04	0.363
Acetate:propionate	4.98	4.83	0.16	0.455

^a^BW bodyweight, DOM digestible organic matter, VFA volatile fatty acids

^b^CON ewes fed basal diet, AL ewes fed basal diet supplemented with allicin

**Table 5:** Effects of supplementary allicin (AL) on ruminal microbial population

Microbial population, per mL of ruminal fluid	Treatments^a^		SEM	*P* value
	CON	AL		
Total bacteria, × 10^10^	7.36	12.10	0.72	<0.001
Protozoans, × 10^7^	7.83	6.64	0.36	0.097
Methanogens, × 10^7^	9.23	4.53	0.79	0.001
*F. succinogenes*, × 10^5^	4.08	9.05	0.66	<0.001
*R. flavefaciens*, × 10^8^	4.18	6.84	0.43	0.001
*R. albus*, × 10^7^	6.44	6.79	0.40	0.675
*B. fibrisolvens*, × 10^9^	9.71	15.20	0.89	0.001

^a^CON ewes fed basal diet, AL ewes fed basal diet supplemented with allicin

Supplementary AL increased the total bacteria (*P* < 0.001), (Table 5), decreased the population of methanogens (*P* = 0.001), and tended to decrease the population of protozoans (*P* = 0.097). Populations of *F. succinogenes* (*P* < 0.001), *R. flavefaciens* (*P* = 0.001), and *B. fibrisolvens* (*P* = 0.001) were significantly increased by supplementation of AL, while no effect of AL was found on the population of *R. albus* (*P* = 0.675).

## Discussion

The current study found that supplementation of AL increased the apparent digestibility of OM, N, NDF, and ADF. It is reported that AL is very unstable and quickly changes into a series of other sulfur-containing compounds such as DADS [23]. In a related study, it was reported that supplementation of DADS at 2 g/kg of diet improved the apparent digestibility of OM and NDF in sheep [10]. Kamruzzaman et al. [24] also reported that replacing 10 % of hay by garlic leaf, which retains the same bioactive components as the garlic bulb, could increase N digestibility in sheep. The increase in nutrient digestibility could be explained by the increase in the populations of cellulolytic bacteria (*F. succinogenes*, *R. flavefaciens*, and *B. fibrisolvens*) in the rumen as observed in current study, which in turn improved the utilization of dietary fiber and provided more carbohydrates to microbes.

Nitrogen retention is considered an index of protein status in ruminants. The lower N output in feces in the AL group is consistent with the higher digestibility of dietary N, suggesting an improved utilization of dietary N. Urinary N output was similar between the two groups in current study. When scaled to metabolic bodyweight, however, a significant decrease in the AL group was observed (0.61 vs 0.69 g/kg BW^0.75^/d, *P* < 0.05). A reduction of urinary N excretion is desirable, as urinary N causes more waste and pollution to the environment than fecal N, as feces could be utilized for crop production when used as a manure [25]. Supplementary AL tended to increase both N retention and the ratio of N retention/N intake. The insignificant N retention could be due to the dosage of AL used in the current study. As reported by Wanapat et al. [9], supplementation of garlic powder at 40 g/day did not affect N retention, but at 120 g/day did improve N retention in steers.

The current study found that supplementation of AL decreased daily methane emissions (L/kg BW^0.75^) or methane output scaled to DOM intake. Previous studies showed that methane production was suppressed *in vitro* by garlic oil [26, 27]. Similar to our results, Klevenhusen et al. [10] found a decrease in methane output scaled to digested NDF intake when DADS was supplemented and Patra et al. [28] found that supplementary *Allium sativum* tended to reduce methane output scaled to digested DM intake by sheep. Zhu et al. [29] found that the final step of biohydrogenation was interrupted in the rumen of goats by infusion garlic oil; this may be related to its antibacterial activity. All these *in vitro* and *in vivo* results suggest that garlic components are effective in reducing methane emissions. This effect may be due to the reduction of methanogen or protozoan populations, as observed in current study. It has also been reported that endo- and ecto-symbiotic methanogens of protozoans could contribute up to 25 % of rumen fluid methane emissions in sheep [30].

Supplementary AL decreased the ruminal concentration of ammonia, but increased that of total VFA, which is similar to results reported by Cardozo et al. [31] and Klevenhusen et al. [10], who supplemented various garlic components *in vitro* and in sheep diets, respectively. Again, those results could reflect enhanced utilization of dietary fibrous components by ruminal microbes as the population of *R. flavefaciens* increased. The change in the molar proportion of acetate, isobutyrate, and butyrate suggested that supplementary AL might affect rumen fermentation patterns by changing microbial populations. Reports of the effect of garlic components on ruminal VFA were inconsistent. Concentration of VFA and the molar proportion of acetate decreased, but the molar proportion of propionate and butyrate increased [26]; concentration of VFA and the molar proportion of propionate increased [9]; and neither the total concentration nor the molar proportion of VFA was affected by the additives [10]. The experimental differences among these results could be related to the experimental diets and dosage of the plant extract used.

Although not significant, the population of protozoans tended to decrease in response to supplementary AL. The effect of garlic by-products on protozoan numbers differed in different studies. Reuter et al. [32] reported that garlic extracts are effective against a host of protozoans. Kongmun et al. [33] investigated the effect of garlic powder on *in vitro* fermentation and found a reduced protozoan count. Anassori et al. [34] found that supplementing a basal diet with raw garlic or garlic oil effectively reduced number of total protozoans in sheep. Those discrepancies could be attributed to factors such as specific diet and supplementary dosage.

In the current study, supplementary AL decreased the population of methanogens by about 104 %. Most studies of the effect of garlic components on the population of methanogens were conducted *in vitro*. Chaves et al. [27] reported that supplementing garlic oil decreased methanogenic activities of mixed ruminal bacteria. More recently, Patra and Yu [35] reported that garlic oil could reduce the abundance of archaea. Observations of the reduction of methanogens in the current study coincide with those of *in vitro* results. The reduction of methanogens could be directly due to the inhibitive effect of garlic components. In addition, the decreased population of protozoans could also be responsible for the reduction in methanogens, as the total methanogen population declined in absolute number as well as in proportion to the total bacterial population in the absence of protozoans [36].

In our study, we quantified four main cellulolytic bacteria using a q-PCR system and observed significant increases in the populations of *F. succinogenes*, *R. flavefaciens*, and *B. fibrisolvens* in ewes supplemented with AL. Wanapat et al. [9] reported that supplementation of garlic powder did not affect the population of amylolytic or cellulolytic bacteria. Patra and Yu [35], however, reported that garlic oil effectively reduced the *in vitro* abundance of *F. succinogenes, R. flavefaciens,* and *R. albus* without affecting that of total bacteria. It should be noted that although garlic has been proven effective against some gram-negative or gram-positive bacteria, it is not a broad-spectrum microbial inhibitor [37]. In addition, rumen is such a complicated system that *in vitro* studies could not completely reflect the situation in rumen. The increase in the population of those three cellulolytic bacteria could be more probably explained by the reduced populations of the protozoans that engulf bacteria. To our knowledge, there has been no study on the effect of garlic-related compounds on ruminal microbial flora in sheep; further study is needed to prove the effectiveness of AL in manipulating certain microbes.

## Conclusions

Dietary supplementation of AL at 2.0 g/head·day effectively enhanced OM, N, NDF, and ADF digestibility and reduced daily methane emissions (L/kg BW^0.75^) in ewes, probably by decreasing the population of ruminal protozoans and methanogens.
